# Mechanical Properties and Fracture Analysis of Advanced Nickel-Based Nanomembranes

**DOI:** 10.3390/ma18091961

**Published:** 2025-04-25

**Authors:** Janik Marius Lück, Joachim Rösler

**Affiliations:** Institut für Werkstoffe, Technische Universität Braunschweig, Hans-Sommer-Straße 5, 38106 Braunschweig, Germany; j.roesler@tu-braunschweig.de

**Keywords:** CMSX-4, superalloy membranes, creep test, fracture analysis, rafting, EBSD

## Abstract

Nanoporous membranes based on the single crystalline nickel-based superalloy CMSX-4 are a promising class of materials for membranes, especially for use in premix membrane emulsification. In addition to the pore size, the strength and stability of the membrane structure are key factors for subsequent use. The production of the membranes is based on the directional coarsening of the γ/γ′-microstructure by creep deformation, in which the material is subjected to a tensile load at high temperatures so that a bicontinuous network of the γ- and γ′-phase is formed. The subsequent dissolution of the γ-phase leaves a network of γ′-phase, which can be used as a membrane structure; the former γ-matrix channels now serve as pores. Previous investigations focusing on the evolution of the microstructure during membrane fabrication found that a particularly small pore size can be achieved when the creep deformation temperature is lowered from 1000 °C to 950 °C while increasing the stress from 170 MPa to 250 MPa. This study will now investigate the strength and fracture behaviour of membranes produced by these improved parameters. For this purpose, four creep states with creep strains between 1.3% and 5.7% are investigated in tensile tests at room temperature, with the load being applied perpendicular and parallel to the raft structure. The results show that the strength of nanomembranes during perpendicular loading essentially depends on the cross-linking between γ′-rafts. Generally, an increase in creep strain leads to an increase of the cross-linking resulting in higher tensile strength. During parallel loading, γ′-inhomogeneities play an important role resulting in a loss of strength. The analysis of the fracture surfaces and evaluation of EBSD measurements reveal an insufficient cross-linking between dendrites and around γ′-inhomogeneities, leading to preferred crack paths. Therefore, the differences in orientation within the single crystal play a key role in the strength of the nanomembranes.

## 1. Introduction

As demonstrated by Kohnke et al. [1], nanomembranes based on the single crystalline nickel-based superalloy CMSX-4 are suitable for use in premix membrane emulsification. This process can be used, for example, to produce nanoemulsions in pharmaceutical applications. The production of the nanomembranes begins with (i) adjusting a suitable γ/γ′-microstructure via a heat treatment consisting of a solution and precipitation heat treatment. A raft structure is then created using (ii) directional coarsening in a creep process at high temperatures and constant tensile stress. As a bicontinuous network of γ- and γ′-phase is formed, the γ-matrix can be dissolved in the next step (iii) using selective phase extraction, leaving a membrane-like structure. In a first study by the authors [2], the heat treatment for adjusting the γ/γ′-precipitate structure was already optimised in such a way that the γ′-precipitates and γ-matrix channels are as small as possible. The optimised precipitation heat treatment was carried out for 30 min at a temperature of 1140 °C and achieved a γ′-particle size of 224 ± 52 nm and a γ-matrix channel width of 35 ± 19 nm [3]. On this basis, it was shown in a subsequent study by the authors [3] that the combination of a temperature of 950 °C and a stress of 250 MPa leads to the best results, achieving a possible pore width of about 119 ± 43 nm after 310 h with complete crosslinking in dendritic areas.

Based on the elaborated results, in this study, the mechanical properties are analysed as a function of the creep strain obtained by creep testing at 950 °C/250 MPa. For this purpose, four creep strain conditions between 1.3% and 5.7% are investigated in tensile tests at ambient temperature to enable a comparison with results obtained by Rösler et al. [4] on previous membranes with a pore size of about 300 nm to 400 nm. These membranes originate from creep tests at 1000 °C/170 MPa with creep strains between 1.5% and 11.5% and represent the state-of-the-art membranes based on CMSX-4. A distinction is made between loading perpendicular and parallel to the raft structure. The subsequent fracture surface analysis should provide information about the failure mechanisms. In order to be able to investigate the γ′-inhomogeneities already observed by Rösler et al. [4] and their influence on the strength of the nanomembranes under parallel loading in more detail, electron backscatter diffraction (EBSD) measurements were also carried out at selected points of interest. These will be used to investigate the possible influence of an angular deviation between dendrites. This leads to the following research questions:

1. How does a change in the process parameters from 1000 °C/170 MPa to 950 °C/250 MPa affect the strength of the nickel-based nanomembranes?
2. How do the fracture mechanisms differ between perpendicular and parallel loading of the raft structure?
3. To what extent do inhomogeneities in the microstructure influence the strength under parallel loading of the raft structure and what could be the possible mechanism of origin?

## 2. Materials and Methods

### 2.1. Creep Test Samples and Sample Preparation

Four single crystals were used to investigate the mechanical properties of membranes based on the Ni superalloy CMSX-4 [5]. The single crystal rods were melted by the company Access Aachen e.V., Aachen, Germany using the Bridgman process with a length of 210 mm and a diameter of 21 mm. The angular deviation θ of the [001] crystal orientation from the rod’ centre line was determined by XRD Eigenmann GmbH, Schnaittach-Hormersdorf, Germany using the Laue method and was less than 11° for all single crystals used. Before the single crystals were prepared for further use in the corresponding sample form, a heat treatment was carried out. This was based on the results of previous investigations by the authors [2] and consists of the following solution and precipitation heat treatment: 1277 °C/2 h + 1288 °C/3 h + 1296 °C/3 h + 1304 °C/2 h + 1313 °C/2 h + 1316 °C/2 h + 1318 °C/2 h + 1321 °C/2 h + AC to RT + 1140 °C/30 min + AC to RT (AC, air cooling; RT, room temperature). Creep samples were then produced from the single crystals. The creep samples have an M16 standard thread at the ends and a rectangular cross-section in the centre, which is required for later membrane production. Following sample preparation, the manufactured samples were subjected to a constant load in an electromechanical test rig from WALTER+BAI, Löhningen, Switzerland at a temperature of 950 °C, so that a stress of 250 MPa prevailed in the relevant cross-section over the entire duration of the test. The end of the creep tests was determined using an elongation criterion so that a permanent creep strain between 1.3% and 5.7% could be set. After removal of the samples from the testing setup, the permanent creep strain *ε_pl_* was determined using indentations at both ends of the rectangular cross section. The values were chosen so that a meaningful comparison could be made with the results of Rösler et al. [4], who investigated membranes based on a creep temperature of 1000 °C and a stress of 170 MPa. Furthermore, the selected strain range takes into account results from previous studies by the authors in which a minimum achievable γ-channel width was investigated [3]. A summary of the data on the single crystals and creep parameters used can be found in Table 1. For completeness, it should be noted that the creep test of the single crystal with the specimen-ID CMSX-4/3.9/395 was interrupted two times after approx. 24 h and 355 h due to a software error and a fracture of the pull rod. It should also be noted that the single crystal CMSX-4/5.7/347 has a stray grain, which will be considered in the later interpretation and discussion of the results. Accordingly, samples taken from this single crystal will be explicitly labelled as “single crystal with stray grain” in the following where appropriate.

**Figure 1:**
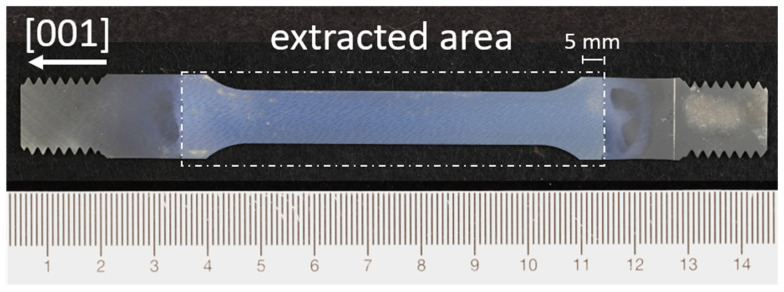
Flat tensile specimen of shape E in the extracted state. The parallel length is at least 55 mm, 5 mm of extracted area were added on each side of the tapered section to force specimen failure in the measuring range.

Following the creep tests, creep samples were further processed into sheets with a thickness of 0.45 mm, whereby the creep sample was cut parallel to the rod axis and thus parallel to the [001] sample orientation using spark erosion. Depending on the thickness of the creep sample, 8 to 9 sheets could be produced, which were then used for the tensile tests. For subsequent membranes, which were loaded perpendicular to the raft structure, 4 sheets of each creep sample were prefabricated using a cutting process in accordance with the tensile specimen shown in Figure 1. The shape E flat tensile specimen defined in DIN 50125 was selected as a guideline; in all cases, the parallel length was at least 55 mm. Furthermore, the material area beyond the taper was also extracted with a length of approx. 5 mm in order to favour a fracture in the parallel length and not a fracture in the transition area between the solid material and the membrane. Limiting the extraction to the specified area was achieved by masking off the area not to be extracted using adhesive tape [6] originally intended for anodising processes. Later membranes, which were loaded parallel to the raft structure, were eroded from the previously eroded sheets into discs with a diameter of 15.29 mm. In contrast to the tensile tests with perpendicular loading of the raft structure, the round samples were not masked with adhesive tape, and thus were not solid in the clamping area after extraction. In order to produce a membrane from the existing sheets and discs, selective phase extraction was used as follows: The samples were first ground to the desired thickness using a grit size of P2500. The γ-matrix was then dissolved in an electrolyte consisting of 800 mL H_2_O, 8 g (NH_4_)_2_SO_4_, and 8 g C_6_H_8_O_7_ at a constant voltage of 1.3 V [7,8]. Once the extraction was complete, the samples were cleaned for further use in an ultrasonic bath in ethanol for 10 min. During the production of the tensile test specimens, it was found that the single crystal CMSX-4/5.7/347 contains a stray grain. This has to be taken into consideration when interpreting the results later.

### 2.2. Tensile Tests

**Figure 2:**
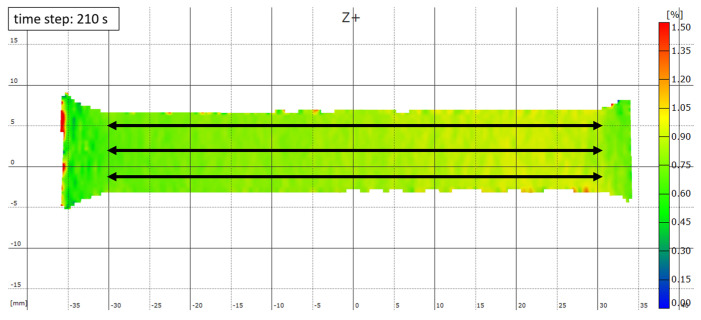
Exemplary illustration of the strain analysis of a tensile specimen after a test duration of 210 s in the test using the ARAMIS software. Three arrows visualise the strain measurement, the colour coding illustrates the strain.

To investigate the mechanical properties, tensile tests were carried out using an electromechanical tensile testing machine from ZWICKROELL, Ulm, Germany at RT and at a speed of 0.2 mm min-1 [4]. Tensile test specimens, such as those shown in Figure 1, were loaded perpendicular to the raft structure in the tensile test. To measure the strain, an optical measuring system gom ARAMIS 5M from the manufacturer GOM GmbH, Braunschweig, Germany was used in addition to the standard recording of the crosshead travel by the tensile testing machine. For this purpose, one side of the tensile specimen was provided with a fine reference pattern, which the system can use to measure the superficial change in the position of individual points during the tensile test. Measurements were taken with a temporal resolution of 1 Hz. In order to determine the change in length of the parallel gauge length, a total of six measurement points were defined at the left and right end of the gauge length—two each in the top, middle, and bottom part of the samples. Linking two of these points across the gauge length results in three independent values of change in length between the linked points. The resulting values for each time step are then averaged to calculate the resulting strain. Figure 2 shows an example of how the length change is measured using the ARAMIS v6.1 software at the 210 s time step of a tensile test. Additionally, the strain is shown as colour coding from 0% (blue) to 1.5% (red), which allows for determining not only the total strain but also strain peaks and local differences. Using the recorded force by the ZWICKROELL universal testing machine and the strain measured with the GOM system, the elastic stiffness E* was calculated during loading of the sample using the strains at stress levels of 10 and 40 MPa and assuming linear behaviour between strain and stress, which is well justified, as shown later.

As mentioned above, round specimens with a diameter of 15.29 mm were used for tensile testing of membranes parallel to their raft structure. Due to the small size of the membrane and the tensile tests carried out outside the standards, the GOM system was not used here. Due to the inaccuracies in the traverse path, the strains for these tests were not considered in this investigation. To estimate the cross-sectional area in which the fracture occurs, we measured the thickness *t* of the membranes in a ZEISS LEO 1550 GEMINI SEM, ZEISS, Oberkochen, Germany while the width *w* perpendicular to the loading direction was estimated with the help of photographs and IMAGEJ v1.54c [9]. Since the crack path was generally not oriented perpendicular to the applied stress, *w* changes along the crack path. Consequently, we calculated *R_m_*_,*min*_ and *R_m_*_,*max*_ as lower and upper bounds of the tensile strength using the widest and smallest cross-sectional area along the fracture path, respectively. For tensile tests in which the load was applied perpendicular to the raft structure, four tensile specimens were tested per creep state. In the case of loading parallel to the raft structure, there were two specimens each for creep strains of 1.3% and 5.7% and one specimen each for creep strains of 2.7% and 3.9%.

### 2.3. Microstructure Analysis

Scanning electron micrographs of the membrane surfaces, taken with a ZEISS LEO 1550 GEMINI, were used to measure the pore size in dendritic and interdendritic areas. In dendritic and interdendritic areas, 5 images each were taken at a magnification of 8k and then cropped to a size of 688 × 688 pixels. The IMAGEJ v1.54c software [9] with the Trainable Weka Segmentation plug-in [10] was used to binarise the images. The images were then analysed using the line intersection method and plotted polar in a structural ellipse where lines parallel and perpendicular to the raft orientation correspond to 0° and 90°, respectively. A detailed description of the procedure is given in Lück et al. [3] and Rösler et al. [4].

### 2.4. Fracture Analysis

The fracture surface analysis was carried out using selected fragments of the membrane samples. A ZEISS LEO 1550 GEMINI was used. To analyse the fracture surfaces, the samples were first cleaned in an ultrasonic bath in ethanol to remove the reference pattern originating from the GOM measurement and prevent contamination of the device.

### 2.5. Electron Backscatter Diffraction—EBSD

The investigation of γ′-inhomogeneities is carried out using an EBSD system from EDAX, Mahwah, NJ, USA, which is installed on a FEI HELIOS NANOLAB 650 DUAL BEAM system, Thermo Fisher Scientific, Hillsboro, OR, USA. Version 4.6.2 of the TEAM software and version 7 of the TSL OIM Analysis software were used to generate and analyse the data. The EBSD measurements were carried out with an acceleration voltage of 20 kV and a current of 6.4 nA. The step size varied depending on the selected magnification, common values used are 0.10 µm to 0.25 µm. After data generation, the data sets were also cleaned up to eliminate any misindexing. To analyse the measurements with regard to the deviations of the orientations in the crystal structure, the misorientation profile was determined and visualised using the misorientation plot function. The orientation of each measurement point along a vector is related to the orientation of the first measurement point so that the change in orientation from point to point can be visualised. The colour coding shown in the EBSD measurements also refers to a reference value, which was determined manually for each measurement at an appropriate distance from the γ′-inhomogeneities under consideration. The reference point is marked with a white dot in the EBSD map. The coding range was then selected so that essentially all measurement points were included. The coding range is specified in degrees, and the coding follows a colour gradient from blue to red so that small deviations are shown in shades of blue and larges ones in shades of red. Measuring points with deviations beyond this are shown in white.

## 3. Results

### 3.1. Pore Size and Structural Comparison

**Figure 3:**
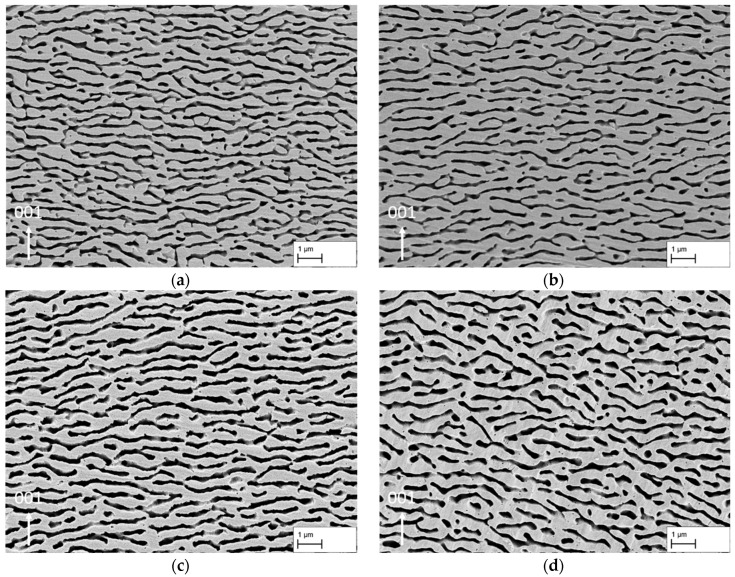
Comparison of the structures of the membranes used in this study with creep strains of (**a**) 1.3%, (**b**) 2.7%, (**c**) 3.9%, and (**d**) 5.7%. The images show dendritic areas of the membranes.

Figure 3a–d shows the membrane structures used in this study. The images are examples of dendritic areas of the membranes with a continuous and homogeneous raft structure. In Figure 3d only, the structure gives the overall impression that larger portions of the γ′-ligaments and γ-channels are slightly tilted relative to the horizontal axis, reflecting the general raft orientation. This only occurs sporadically in the other structures. It can also be seen that the structure in Figure 3d has a coarser appearance and that the γ-pores appear larger.

**Figure 4:**
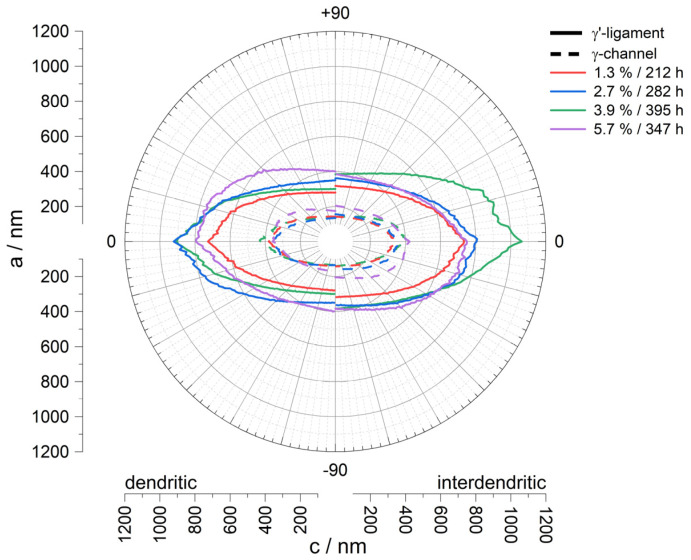
Visualisation of the results of the line section method in a structural ellipsis. The corresponding values were plotted for dendritic (**left**) and interdendritic (**right**) regions.

This observation is supported by the results of the line intersection method summarised in Table 2. While the value a_d,γ-channel_, which is defined as γ-pore width in this study in the following, shows hardly any change for the membranes, with a creep strain of 1.3% (142 ± 44 nm), 2.7% (134 ± 42 nm), and 3.9% (134 ± 39 nm), the value for the creep strain of 5.7% (174 ± 55 nm) increases. Furthermore, the values for the length of the γ-pores show that a maximum pore length is reached at a creep strain of 3.9% (427 ± 351 nm) before the value for the creep strain of 5.7% (358 ± 270 nm) falls to a minimum. Note, however, that tilting of the pores relative to the horizontal axis contributes to this decline. A consideration of the ratio of pore length to width in dendritic areas ((c/a)_d,γ-channel_) at this point illustrates only minor changes from 2.67 for 1.3% to 2.80 for 2.7% and to 3.19 for 3.9%, while the aspect ratio drops to 2.06 for 5.83%. A similar trend can also be observed in the γ′ ligament length, which initially increases from 1.3% (726 ± 596 nm) to 2.7% (921 ± 725 nm), remains at a similar level for 3.9% (904 ± 755 nm), and decreases again for 5.7% (794 ± 588 nm). Figure 4 illustrates the above-described results from the line intersection method for dendritic (left) and interdendritic (right) areas using a structural ellipsis. A set of lines rotates from 90° (lines perpendicular to the horizontal axis) to 0° (lines parallel to the horizontal axis) during the measurement. The resulting intersect lengths are then plotted as a function of the rotation angle to visualise the pore shape. In summary, it can be said that the membrane structures for creep strains of 1.3%, 2.7%, and 3.9% are very similar in terms of pore width, but there are minor differences in the γ′ ligament length and pore length. Only the structure with a creep strain of 5.7% slightly differs from the other membranes due to the tilting of the pore structure relative to the horizontal axis and the slightly larger pores.

### 3.2. Tensile Test Perpendicular to the γ′-Raft Structure

**Figure 5:**
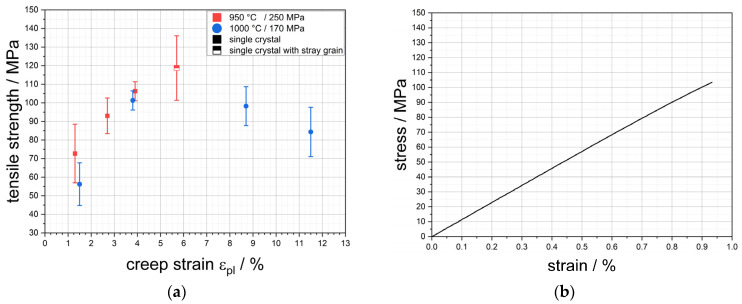
(**a**) Results of the tensile tests perpendicular to the raft structure. The strength values from Rösler et al. [4] for membranes with the process parameters 1000 °C/170 MPa and creep strains between 1.5% and 11.5% are also given to allow for a comparison between the process parameters and the resulting properties of the membranes. (**b**) Exemplary stress–strain curve of the sample CMSX-4/3.9/395-1.

The results of the tensile tests for the load perpendicular to the raft structure are shown in Figure 5a. It illustrates the tensile strength *R_m_* as a function of the creep strain *ε_pl_*. The tensile strengths resulting from this investigation are shown in red, while the results of Rösler et al. [4] are shown in blue for comparison. Firstly, it can be seen that the global trend in tensile strength with increasing creep strain is similar for membranes with the parameter combination 950 °C/250 MPa and 1000 °C/170 MPa. Nevertheless, it is clear that membranes with the combination 950 °C/250 MPa have a higher tensile strength, which is evident in both the lower and upper creep strain range. Despite the presence of a stray grain, tensile strengths of approx. 119 MPa were achieved on average for a creep strain of 5.7%. The data summarised in Table 3 also show that a tensile strength of 137 MPa could be measured for the CMSX-4/5.7/347-1 sample, which corresponds to the highest measured value for this load direction. Furthermore, the data from the GOM system show that there is no clear dependence of the elongation at fracture on the creep strain. Overall, elongations at fracture occur between 0.6% (CMSX-4/1.3/212/1) and 1.0% (CMSX-4/3.9/395-7). Figure 5b shows the stress–strain curve for the specimen CMSX-4/3.9/395-1 illustrating the nearly linear elastic behaviour of the CMSX-4 membranes until fracture.

A comparison of the elastic stiffness E* shows that a trend of increasing stiffness of the membranes can be observed with increasing creep strain. It increases from 8.9 ± 0.9 GPa at a creep strain of 1.3% to 15.9 ± 0.8 GPa at 5.7% creep strain, even though there is a slight drop from 13.9 ± 0.7 GPa at 2.7% to 11.6 ± 0.3 GPa at 3.9% creep strain.

**Figure 6:**
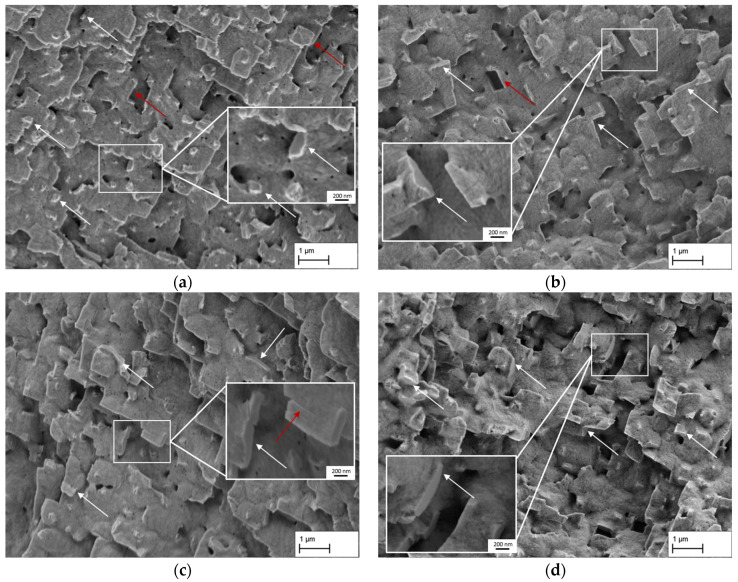
SEM images of dendritic areas of the fracture surfaces of tensile specimens with different creep strains: (**a**) 2.7% (CMSX-4/2.7/282-3), (**b**) 2.7% (CMSX-4/2.7/282-7), (**c**) 3.9% (CMSX-4/3.9/395-7), and (**d**) 5.7% (CMSX-4/5.7/347-1).

Figure 6 shows different images from the fracture surface in the sample centre area of selected tensile test specimens to investigate the cross-linking of the membrane structure. In Figure 6a the fracture surface from CMSX-4/2.7/282-3 is displayed with a tensile strength of 82 MPa at fracture. The platelike structure reveals several cross-linking points—some of which are exemplarily marked with white arrows—that connected the two fracture surfaces prior to fracture. The additional magnification of one cross-linking point also indicates that the rafts themselves fail with a smooth fracture surface without dimples. Additionally, the red arrows mark exemplary points where failure along preferred orientations are visible. In comparison, Figure 6b shows the fracture surface of the specimen CMSX-4/2.7/282-7 with a tensile strength of 104 MPa. The platelike structure of the membrane appears more regular with an increased homogeneity compared to Figure 6a. The fractured areas that served as cross-links prior to fracture appear to be somewhat larger and are more easily discernible. The magnified area shows two smooth, parallel fracture surfaces where the ligament in between was apparently sheared off. Other fracture points where the raft structure failed are marked by additional white and red arrows. In all cases, shear failure along preferred orientations becomes apparent. A particularly prominent example is shown by the red arrow where the connecting ligament was apparently ripped off by shear fracture, leaving a hole in the form of a rhomboid behind. Figure 6c shows the fracture surface of the sample CMSX-4/3.9/395-7 with a tensile strength of 113 MPa. The overall appearance is comparable to the fracture surface in Figure 6b with a platelike structure. Due to the slightly angled view on the surface, the smooth fracture surfaces are well visible (see arrows and the magnified inset). The red arrow in the inset reveals a location where shearing of a γ′-platelet started but did not continue to final failure. Overall, the dimensions of the rafts and visible fractured areas are slightly increased. Figure 6d shows the fracture surface of CMSX-4/5.7/347-1 with a tensile strength of 137 MPa. It reveals a different microstructure, with the platelike appearance being less pronounced. The structure looks more three-dimensional and conveys a well cross-linked impression. Nevertheless, shear failure along preferred orientations is still the dominant fracture mechanism (see white arrows and inset). Overall, it is noteworthy that the fracture surfaces are oriented with their normal directions mostly perpendicular to the loading direction and not parallel irrespective of the prior creep strain.

**Figure 7:**
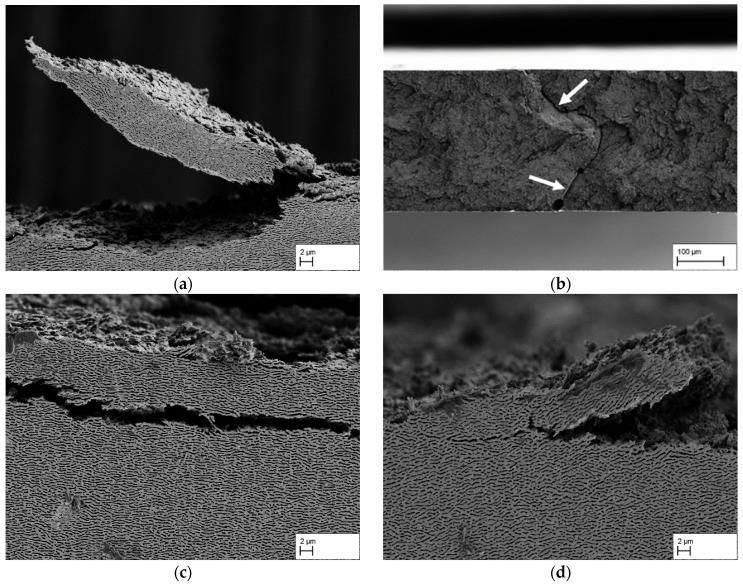
Selection of fracture patterns of tensile specimens with the load direction perpendicular to the raft direction. Typical fracture surface characteristics are shown as they can be found in the four different creep states: (**a**) 1.3% (CMSX-4/1.3/212-5), (**b**) 2.7% (CMSX-4/2.7/282-5), (**c**) 3.9% (CMSX-4/3.9/395-3), and (**d**) 5.7% (CMSX-4/5.7/347-1).

Figure 7 shows side and top views of fractured areas for the tensile specimens that were loaded perpendicular to the raft direction. Although the tensile specimens macroscopically indicate absence of plastic deformation, microscopically it can be clearly seen that there is plastic deformation of the rafts and the raft structure. This can be observed, as shown in Figure 7a for specimen CMSX-4/1.3/212-5, in a plastic deformation and an upward bending of larger areas of the raft structure. Particularly on the right-hand side of the bent-up raft structure, it is furthermore clear that plastic deformation and failure of individual rafts also occurs. This kind of characteristic is also present in Figure 7d for a creep strain of 5.7%. Figure 7b shows a top view of the fracture surface of the CMSX-4/2.7/282-3 tensile specimen, which appears very fissured overall. The fracture surface does not run continuously on one plane but has many differences in height, which could be described as a kind of facet. The gap running through the centre of the image (see arrows), which divides the fracture surface into two halves, is also remarkable. Figure 7c shows a crack through the raft structure slightly beneath the fracture surface, which is typical for the fracture zone right at the edge of the fracture surface when looking from a side view. To summarise the results, it can be said that the fracture surface in the centre of the specimens differs in dependence of the creep strain. However, even at a given creep strain, major differences regarding the cross-linking can be observed that result in different tensile strength. Plastic deformation of the raft can be found in all specimens, independently of the creep strain or tensile strength.

### 3.3. Tensile Tests Parallel to the γ′-Raft Structure

**Figure 8:**
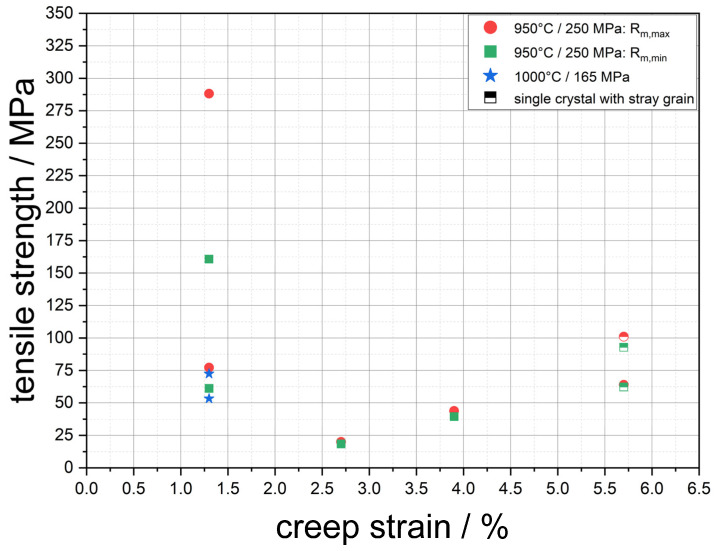
Results of the tensile tests parallel to the raft structure. The strength values from Rösler et al. [4] for membranes with the process parameters 1000 °C/165 MPa and a creep strain of 1.3% are also given.

Figure 8 shows the results of the tensile tests in which the stress was applied parallel to the raft structure. As mentioned above, the lower and upper limits of the tensile strength *R_m_*_,*min*_ and *R_m_*_,*max*_ are plotted against the creep strain for each sample. For the reason of completion, the data are summarised in Table 4. Furthermore, the results from Rösler et al. [4] are also given in Figure 8 to enable a comparison of membranes with different process parameters. The results of the tensile tests show a trend that differs from the previously investigated load direction perpendicular to the rafts. While the CMSX-4/1.3/212-6-LM sample was able to achieve a tensile strength between 160 and 288 MPa, which is well above the values of previous membranes, a second sample CMSX-4/1.3/212-4-R exhibits a tensile strength between 61 and 77 MPa with the same creep strain, which is within the range of previous membranes. An increase in creep strain does not lead to an increase in tensile strength, as expected, but to a decrease to below 20 MPa for a creep strain of 2.7%. With increasing creep strain, the tensile strength increases again, reaching 39 to 43 MPa for the creep strain of 3.9% and 92 to 101 MPa for 5.7%. It is also clear here that within a creep strain, for example 1.3%, there can be a significant range in strength.

**Figure 9:**
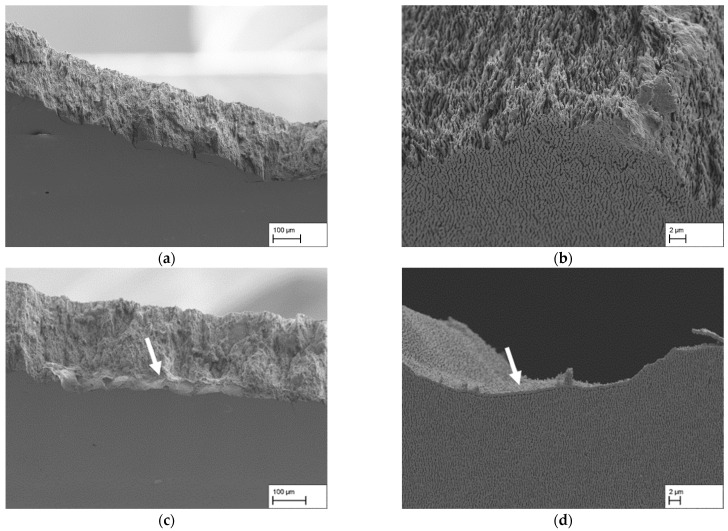
Fracture patterns of the tensile test specimens with a creep strain of 1.3% and loading direction parallel to the raft structure, (**a**,**b**) CMSX-4/1.3/212-6-LM, (**c**,**d**) CMSX-4/1.3/212-4-R.

As the fracture images for the respective creep strains differ significantly from one another, the individual creep states are considered individually. Figure 9a shows an overview of the fracture surface of the CMSX-4/1.3/212-6-LM sample. The fracture surface is characterised over large areas by the fact that it is very rough and fissured, with no clear indications of weak points. A closer examination in Figure 9b also shows that this is primarily a failure due to plastic deformation and, ultimately, rupture of the individual γ′-rafts. This contrasts with the CMSX-4/1.3/212-4-R sample, which also shows areas with a smooth fracture surface in addition to the fissured areas (see arrow in Figure 9c).

**Figure 10:**
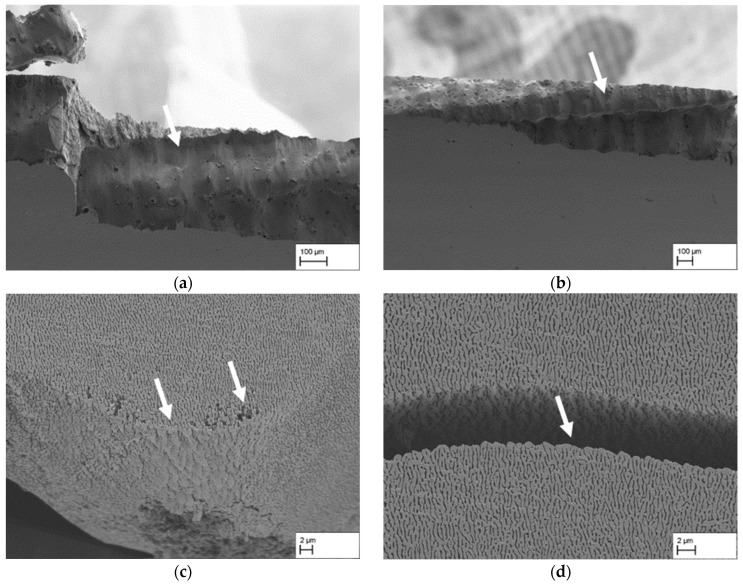
Fracture patterns of a tensile test specimen with a creep strain of 2.7% and loading direction parallel to the raft structure, (**a**–**d**) CMSX-4/2.7/282-R-RM.

A certain similarity to the fracture pattern of the sample CMSX-4/1.3/212-4-R can be seen with increasing creep strain in Figure 10, which shows different areas of the fracture surfaces of the sample CMSX-4/2.7/282-4-RM. The smooth fracture area already shown in Figure 9c can be observed here in Figure 10a and b over long distances of the fracture surface with a wavy profile. Furthermore, the smooth area is not only present at the edge of the fracture surface, but in large parts over the entire fracture surface (see arrows). As an example, Figure 10c,d show that the smooth fracture surface is characterised by larger γ′-precipitates (see left arrow in Figure 10c and arrow in Figure 10d), although there are also areas with cubic γ′-precipitates in the surrounding area (see right arrow in Figure 10c). Along these smooth areas, there are no discernible signs for plastic deformation of the γ′-rafts prior to fracture. This can be particularly well seen in Figure 10d, where both sides of the fracture surface are visible.

**Figure 11:**
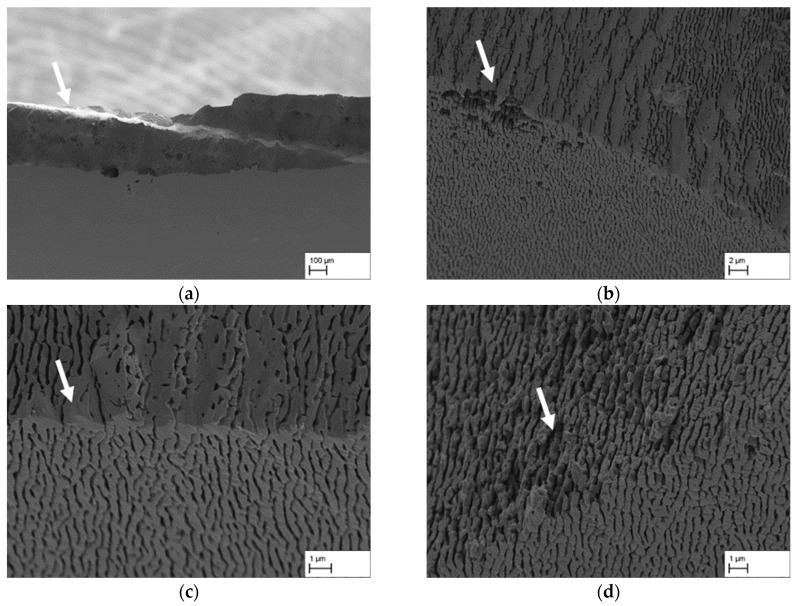
Fracture patterns of a tensile test specimen with a creep strain of 3.9% and loading direction parallel to the raft structure, (**a**–**d**) CMSX-4/3.9/395-4-RM.

The CMSX-4/3.9/395-4-RM sample in Figure 11 shows a fracture pattern similar to that previously observed in the CMSX-4/2.7/282-4-RM sample with a creep strain of 2.7%. As shown in Figure 11a, the fracture surface is characterised by large smooth areas with a wavy profile. Larger γ′-precipitates can again be found in these areas, see Figure 11b,c. Analogous to Figure 10c, cubic precipitates can also be observed for this creep strain, leaving porous areas with missing γ′ precipitates. Figure 11c again clearly shows that a lack of deformation of the γ′-rafts is also characteristic here. Nevertheless, there are also explicit areas in which the raft structure fails due to plastic deformation and direct fracture of the rafts. This becomes clear in Figure 11d (see arrow).

**Figure 12:**
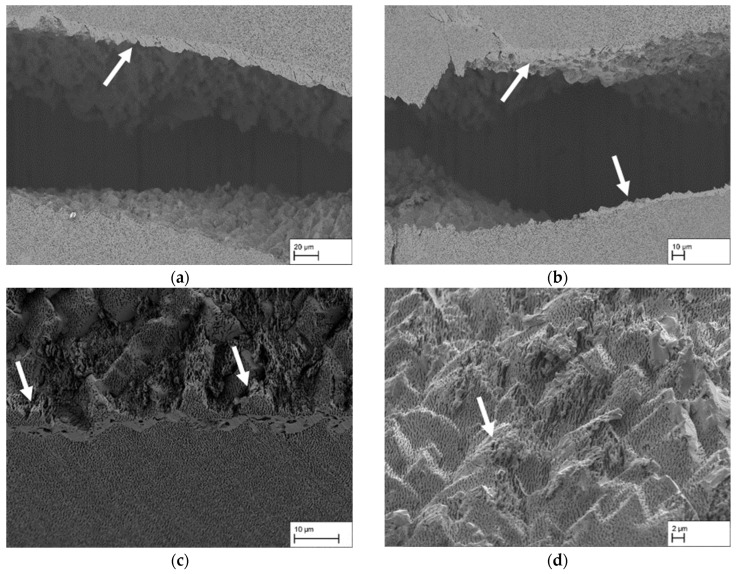
Fracture patterns of the tensile test specimens with a creep strain of 5.7% and loading direction parallel to the raft structure, (**a**–**c**) CMSX-4/5.7/347-8-L, (**d**) CMSX-4/5.7/347-8-LM.

The fracture surfaces of the samples with a creep strain of 5.7% show a preferred crack propagation near the grain boundary. This becomes particularly clear when looking at Figure 12a,b. In addition, it can be seen that the grain boundary is characterised by larger γ′-precipitates, which can also be seen in Figure 12c. A closer look at Figure 12c clearly indicates that the crack does not always run directly along the larger γ′-precipitates, which are located along the grain boundary. Instead, it sometimes runs through the grain (see arrows) which results in local areas where the γ′-rafting structure exhibits plastic deformation on the fracture surface similar to the situation shown in Figure 9a,b.

To summarise, it can be said that the fracture images clearly differ from the previously investigated load direction perpendicular to the raft structure. The proportion of plastic deformation is significantly reduced, especially in case of the samples with a lower tensile strength. Consequently, fracture areas with little to no visible plastic deformation dominate. They appear extremely smooth in many cases and are decorated with large γ′-precipitates. There are also areas where large γ′-precipitates are visible across several γ′-rafts called γ′-bands. Plastic deformation and failure of the actual rafts can be observed above all when the tensile strength is higher. This is particularly evident in the CMSX-4/1.3/212-6-LM sample, which has the highest measured tensile strength of 160 MPa to 288 MPa according to Table 4. In the case of the samples with a grain boundary, a different fracture surface appears along the grain boundary, with both smooth intercrystalline fracture areas along large γ′-precipitates and transcrystalline crack progression through the γ′-raft structure resulting in plastically deformed areas.

### 3.4. EBSD Measurements on γ′-Inhomogeneities

**Figure 13:**
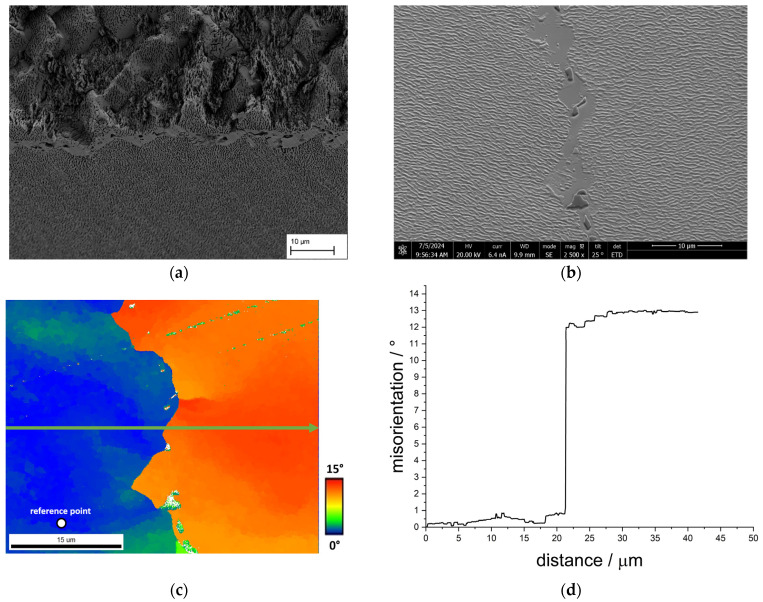
Results of an EBSD measurement using a sample of the single crystal CMSX-4/5.7/347; (**a**) shows an exemplary fracture surface of the grain boundary under investigation from the specimen CMSX-4/5.7/347-8-L, (**b**) shows the area of the EBSD measurement in the SEM image, (**c**) shows an EBSD plot with the arrow indicating the direction of the misorientation plot, and (**d**) the corresponding misorientation plot.

The results of the EBSD measurements are presented in this section. The samples used for this purpose are additional samples from the respective single crystals, as it was not possible to carry out the tensile tests and EBSD measurements on a single sample. The fracture patterns shown in the following are examples of inhomogeneities in fracture areas that are relevant to the failure mechanism. Figure 13a shows again the fracture surface of the sample CMSX-4/5.7/347-8-L with the crack progression in the region of the grain boundary that has already been analysed. Using an additional sample from the same single crystal, Figure 13b details the grain boundary used for orientation measurement. The larger precipitates on the grain boundary, which appear darker than the surrounding γ/γ′-precipitate structure, are noteworthy here. The same area is shown in Figure 13c as an EBSD image, including the point (white dot) where the reference orientation originates from. Areas deviating from this reference orientation are shown in colour using a gradient from blue to red. Consequently, the area to the left of the grain boundary shows only a slight deviation from the reference orientation, while the area to the right of the grain boundary shows a greater deviation. This is also illustrated by the result of the misorientation plot. While the misorientation (viewed from left to right) is less than 1° up to the grain boundary, it rises abruptly to approx. 13° when crossing the grain boundary and then remains almost constant until the end of the measured distance.

**Figure 14:**
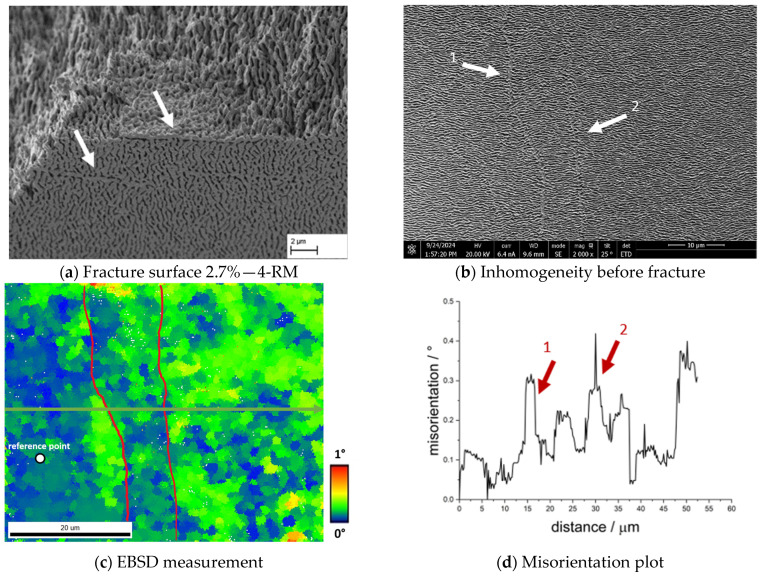
Results of an EBSD measurement using a sample of the single crystal CMSX-4/2.7/282; (**a**) shows an exemplary fracture surface with a γ′-band under investigation from the specimen CMSX-4/2.7/282-4-RM, (**b**) shows the area of the EBSD measurement in the SEM image, (**c**) shows an EBSD plot with the arrow indicating the direction of the misorientation plot, and (**d**) the corresponding misorientation plot.

Figure 14a shows an example of a weakly developed γ′-band at the fracture surface and a γ′-band in the centre of the sample CMSX-4/2.7/282-4-RM (see arrows). The γ′-bands run almost parallel. The upper γ′-band shows a good connection to the underlying structure on the underside, the surface of the γ′-band on the fracture side does not show a smooth but a rough appearance with visible plastic deformation of the γ′-ligaments. Figure 14b shows an SEM image of a comparable area. The adjacent inhomogeneities perpendicular to the raft structure can be clearly observed (see arrows). The corresponding EBSD measurement is shown in Figure 14c, where the red lines indicate the position of the γ′-inhomogeneities. The colour coding already shows that the orientation differences are small and below 1°, as there are predominantly green and blue areas. The misorientation angle along the arrow shown in Figure 14c relative to the orientation at the start of the arrow is plotted in Figure 14d. It changes only slightly and is less than 1° overall. Nevertheless, a trend can be observed in the area of the two inhomogeneities, where there is a slight decrease from approx. 0.3° to approx. 0.1° (arrow 1) followed by an increase to approx. 0.4° (arrow 2).

**Figure 15:**
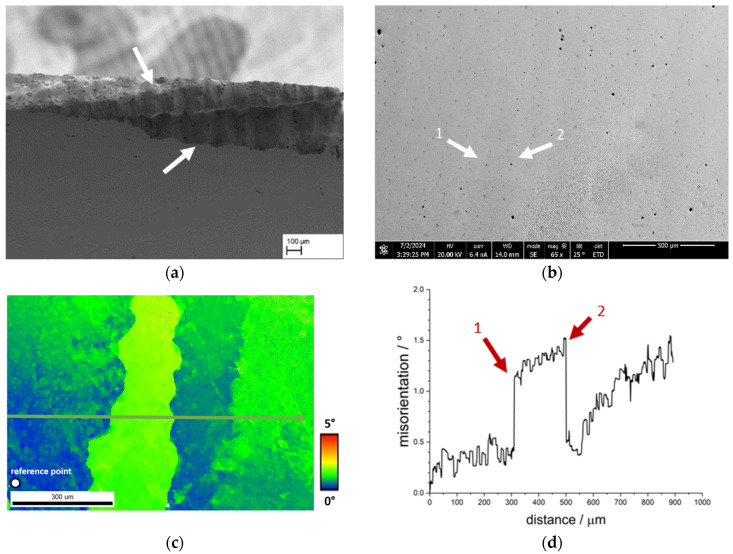
Results of an EBSD measurement using a sample of the single crystal CMSX-4/2.7/282; (**a**) shows an exemplary fracture with a wavy profile from the specimen CMSX-4/2.7/282-4-RM, (**b**) shows the area of the EBSD measurement in the SEM image, (**c**) shows an EBSD plot with the arrow indicating the direction of the misorientation plot, and (**d**) the corresponding misorientation plot.

Figure 15a shows an example of the fracture surface of the sample CMSX-4/2.7/282-4-RM with the typical appearance of the smooth fracture surface with a slightly wavy profile. To get to the bottom of this appearance, an EBSD measurement was carried out at a lower magnification so that possible differences in the orientation of the dendrites become visible. The SEM image associated with the EBSD measurement is shown in Figure 15b,c. The misorientation plot with reference to the first measurement point of the arrow is shown in Figure 15d and graphically represents the differences already observed in the colour coding in Figure 15c. Firstly, it is clear that a deviation of up to approx. 0.5° is already present within the dendrites. As the measuring distance continues, the misorientation increases abruptly to approx. 1.25° at a distance of approx. 300 µm and rises to approx. 1.5°. In the further course, it abruptly decreases again to approx. 0.5° and then increases again to approx. 1.5° over a distance of approx. 300 µm.

**Figure 16:**
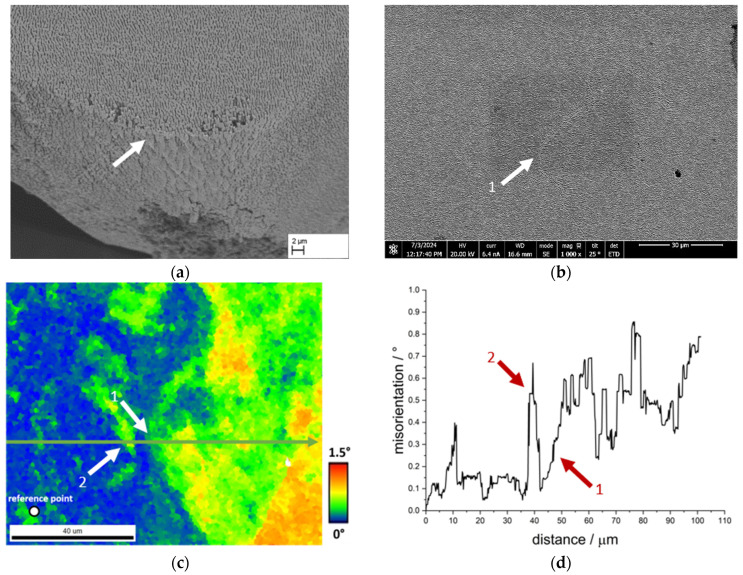
Results of an EBSD measurement using a sample of the single crystal CMSX-4/2.7/282; (**a**) shows an exemplary fracture with γ′-inhomogeneities from specimen CMSX-4/2.7/282-4-RM, (**b**) shows the area of the EBSD measurement in the SEM image, (**c**) shows an EBSD plot with the arrow indicating the direction of the misorientation plot, and (**d**) the corresponding misorientation plot.

Figure 16 again shows an example of the fracture surface of the sample CMSX-4/2.7/282-4-RM already shown in Figure 10c, in which the remarkably large γ′ precipitates are located at the fracture surface, which by itself appears very smooth and free of plastic deformation. The shape and appearance of such an inhomogeneity could be investigated using a sample of the single crystal CMSX-4/2.7/284, and the investigated area in Figure 16b shows a certain similarity with the area of the fracture surface. The corresponding result of the EBSD measurement is shown in Figure 16c. It is already clear from the colour coding that the deviations in this measurement are only slight, which is also confirmed in the course of the misorientation plot in Figure 16d. The plot shows slight orientation differences of up to 0.6° prior to the inhomogeneity (arrow 2) without any visible effect on the raft structure (compare Figure 16b). Passing the inhomogeneity leads to a local increase up to approx. 0.2°, followed by an increasing misorientation with several peaks and drops on an elevated misorientation level.

## 4. Discussion

### 4.1. Pore Structure and Mechanical Strength Perpendicular to the Rafting Structure

By loading the creep specimens along the <001>-direction at a constant temperature of 950 °C and a creep stress of 250 MPa, a directionally coarsened γ/γ′-microstructure with rafts perpendicular to the loading direction was produced (see Figure 3a–d). This is a raft structure of type N, which occurs in alloys with a negative misfit δ. Since the single crystalline nickel-based superalloy CMSX-4 used here has a slightly negative misfit at room temperature, which becomes more negative with increasing temperature, the type N raft structure that occurs is consistent with the previous literature results [4,11,12,13,14,15,16]. The nanoporous membranes produced in this study have a pore size of approx. 140 nm to 170 nm in dendritic areas. Between the achieved creep strains of 1.3% and 3.9%, the pore channel width, i. e., the width of the former γ-channels before extraction of the γ-phase, is almost constant (around 140 nm), considering the standard deviation. Only a further increase in the creep strain to 5.7% leads to an increase in the channel width to approx. 170 nm. The trend for the measured γ-channel widths agrees with the trend of previous studies by Lück et al. [3]. These have shown that a minimum γ-channel width can be achieved by considering the *3ω*_0_ criterion of Epishin et al. [15], but that there must be sufficient creep strain to ensure adequate crosslinking of the γ/γ′-microstructure. A creep strain that is too large can lead to increased coarsening and thus to an increase in the γ-channel width. In this respect, a creep strain of about 4% appears to be a good compromise, combining high strength with narrow pores.

The results of the tensile tests in Figure 5 show an increase in tensile strength with increasing creep strain up to the highest value of 5.7% investigated here. This behaviour is consistent with the previous results of tensile tests of nanoporous membranes based on single crystalline CMSX-4 by Rösler et al. [4], which were produced at a temperature of 1000 °C and a stress of 170 MPa. Furthermore, the results show that an increase in the tensile strength of the membrane structures can be achieved by lowering the creep temperature from 1000 °C to 950 °C and increasing the creep stress from 170 MPa to 250 MPa. According to Müller et al. [17], lowering the temperature leads to an increase in the γ′-volume fraction. The difference of 50 °C between the previous tests by Rösler et al. [4] and the tests in this study can result in a small increase in the volume fraction of approx. 1–2%, which can lead to the small but recognisable difference in the tensile strength. This porosity-dependent behaviour of the strength can also be observed in other structures, which exhibit increasing strength with decreasing porosity [18]. The characteristic of the tensile strength as shown in Figure 5 can be explained with the help of the results from Figure 6. The fracture surface indicates that the tensile strength correlates with the cross-linking of the γ′-rafting. This relation is even present within one creep sample where different tensile test specimens show different fracture surfaces while achieving different tensile strengths. This becomes clear in Figure 6a,b at a creep strain of 2.7% where a less cross-linked structure with smaller cross-linked points and a less dense structure leads to a tensile strength of 82 MPa compared to 104 MPa with more solid cross-linked rafts. Additionally, the structure itself appears more solid and connected. Within the platelike structure, several fractured rafts with a crystallographically preferred orientation of the fracture path are visible, leaving areas with a rhombohedral shape (see red arrow in Figure 6b). It is easy to imagine that the rhombohedral counterpart appears on the other side of the fracture surface (for example see right top arrow in Figure 6a). The orientation of the fracture surfaces of the rafts perpendicular to the loading direction indicates that mainly shear stresses cause the failure of the rafts along preferred crystallographic orientations. This also becomes clear in Figure 6c where shear deformation parallel to the fracture surface of the raft is visible. In general, the results confirm the previous observations of Lück et al. [3] that, although the raft formation process itself appears to be complete early on, it is still necessary to ensure a sufficient creep strain or creep duration so that complete cross-linking of the structure can be achieved. Here, the results demonstrate that the creep strain is more important than the creep time because the single crystal CMSX-4/5.7/347 experienced a shorter creep duration than CMSX-4/3.9/395 but achieves higher strengths.

The general fracture behaviour of the membranes under perpendicular loading of the raft structure, as summarised in Figure 6 and Figure 7, shows a similar picture to that reported by Rösler et al. [4]. Macroscopically, no plastic deformation can be seen, and the stress–strain curve in Figure 5b indicates a linear-elastic behaviour until fracture without clear signs of plastic deformation during loading of the sample. This is also confirmed by the measurement of the fracture strain via the GOM system since it is always very close to the elastic strain at fracture ε_el_ = R_m_/E*. Microscopically, however, clear plastic deformation of the raft structure can be observed by shear deformation (see Figure 6) and by bending of larger raft sections (see Figure 7a,c,d), which is consistent with results obtained in [4]. From this, it can be concluded that the γ′-phase itself is ductile.

### 4.2. Mechanical Strength Parallel to the Raft Structure and Orientation Analysis

As part of this investigation, the raft structure was also loaded parallel to the raft orientation in the tensile test. As it was not possible to produce standardised or standard-like tensile test specimens with a sufficiently large cross-section from the creep specimens used due to material availability, the round specimens intended for later emulsification tests were used. These proved to be quite suitable for the investigation of possible structural defects under parallel loading of the raft structure. It was possible to compare the individual creep states both qualitatively and quantitatively. The main objective that was set at the beginning of this partial investigation, namely a comparison of the creep states and the effects of possible defects, could be achieved. The results in Figure 8 and Table 4 show that there is a wide scatter between the strengths. This applies not only to the individual creep states, but also to samples within a creep state. For example, the range in tensile strength of the two samples with a creep strain of 1.3% is 61–77 MPa and 160–288 MPa, respectively. The values determined by Rösler et al. [4] after a creep deformation of 1.3% at 1000 °C/165MPa were 53 MPa and 73 MPa, respectively, which is why the values determined in this study initially appear to be reasonable. With the aid of the fracture surfaces from Figure 9, Figure 10, Figure 11 and Figure 12, however, a clearer picture emerges, which makes the corresponding values more comprehensible. As Rösler et al. [4] have already established, internal faults and defects play a significantly greater role in the parallel loading of the raft structure than in the loading perpendicular to the raft structure. This is also evident in this investigation. While the sample CMSX-4/1.3/212-6-LM with a tensile strength of 160–288 MPa shows only a small number of defects at the fracture surface and a predominantly plastic failure behaviour, all other fracture surfaces of the various tensile samples examined show significant defects. These include elongated precipitates similar to γ′-bands (see Figure 9d), as well as large areas with smooth precipitate surfaces and little to no visible plastic deformation (see Figure 10c and Figure 11b,c). Images of the fracture surface at lower magnification show, as in Rösler et al. [4], that the crack runs between dendrites so that the shape of the dendrites with their primary and secondary arms is revealed on the fracture surface (see Figure 10a,b and Figure 11a). This indicates that the connectivity in the interdendritic areas has a significant influence on the strength under parallel loading.

Due to these findings, EBSD measurements to determine the misorientation in the area between dendrites and in the vicinity of structural defects will be considered in the following. At this point, it should be pointed out once again that the measurements could not be carried out on the tensile samples, but on separate samples. This must be considered. The grain boundary analysed in Figure 13 shows a misorientation of approximately 13°, and precipitates can also be observed on it. The results indicate that the history of heat treatment consisting of homogenization, precipitation, and thermomechanical heat treatment plays a significant role as the γ′-precipitations form preferably at the grain boundary because nucleation is easier and diffusion faster there [19]. This leads to a pronounced occupation of the grain boundary with γ′-precipitates as observed in Figure 12, which shows similarity to serrated grain boundaries. In polycrystalline Ni-based alloys serrated grain boundaries are used to improve the creep performance and reduce the crack growth rate [20,21,22,23]. During tensile test of the membrane, the grain boundary is a preferred path for the crack. The fracture surface in Figure 12 displays that the crack generally follows the profile of the grain boundary leading to a wavelike fracture surface along the γ′-precipitates. However, due to this pronounced waviness, the crack is also frequently forced into the grain interior, leading to significant plastic deformation there. As a result, the fracture surface displays areas with and without plastic deformation as the crack changes its course from intercrystalline with rather poor connection (no plastic deformation) to transcrystalline with a well cross-linked structure (plastic deformation) and vice versa.

The γ′-bands analysed in Figure 14 with good bonding on one side exhibit a significantly lower misorientation of less than 0.5°. Nevertheless, slight rises and falls can be clearly assigned to the γ′-inhomogeneities. The deviation in the misorientation of up to 1.5° measured in Figure 15c shows the course of a dendrite in the colour coding in a very clear qualitative manner, which shows good agreement with the course of the fracture surface of several samples (see e.g., Figure 11a). This contrasts with the only locally present change in misorientation in Figure 16 (arrow 1), which is approx. 0.2° and increases continuously, followed by several peaks and drops. Here, too, the misorientation plot is in good agreement with the course of the structural inhomogeneity in the SEM image. In comparison to the analysed grain boundary in Figure 13, it is clear that the misorientation in the area of the finer inhomogeneities is significantly lower, but clearly present. Such angular misorientations have been extensively discussed in the literature for single crystalline superalloys. For example, Xu et al. [24] were able to determine similar and even larger misorientations as part of an investigation of a turbine blade made from CMSX-4 using the Bridgman process. According to Xu et al. [24], dendrite fragmentation can lead to sliver defects, a critical misorientation of 3.2° might be the lower boundary for dendrite fragmentation. Below 1°, the authors describe the defect type as deflection, resulting from bending of the dendrites. Aveson et al. [25] differentiate between two types of dendrite bending or deformation: plastic and elastic deformation. In plastic deformation, the dendrite bends until it meets a neighbouring dendrite and a small-angle grain boundary is formed. In the case of elastic deformation, the deformation takes place in front of the interdendritic solidus/liquidus boundary. The interdendritic area solidifies in such a way that the spontaneous elastic deformation of the dendrite is frozen, resulting in a misorientation between the dendritic and interdendritic areas. Furthermore, Aveson et al. [26] were able to observe a spontaneous and localised deformation of the dendrite arms, which influences the growth kinetics and leads to the formation of a small-angle grain boundary at the convergence surfaces of the secondary dendrite arms. Again, it is concluded that sliver grains are formed during solidification in regions with higher thermal stresses as a result of deformation in the mushy zone. Yu et al. [27] were also able to show in creep tests with CMSX-4, which exhibited sliver grains, that the rafting structure is interrupted in the region of the small-angle grain boundary, which essentially has a negative effect on the creep life.

The results in the literature show that local deviations can occur between two dendrites. Below 1° misorientation, the effects on the microstructure appear to be small, which is confirmed, for example, by the measurement of the γ′-inhomogeneity in Figure 14c. This type of γ′-inhomogeneity is present for example at the fracture surface in Figure 14a where the connection is sufficient on at least one side of the γ′-inhomogeneity. Furthermore, the length of the γ′-band is limited to a comparatively small area, so that the effects on the membrane strength can be categorised as low. The results in Figure 16 are different: although only a small misorientation was measured in a comparable EBSD measurement, the fracture pattern shows an enormous effect on the strength. In addition, it is not localised areas that are affected here, but large areas. At this point, a combination of two effects is conceivable. One effect concerns the different rafting behaviour in dendritic and interdendritic areas due to micro-segregation. According to Reed et al. [28], the Orowan stress increases with decreasing γ-channel width. Since the γ-channels are larger in dendritic areas than in interdendritic areas, the stress required for the raft formation process is greater in interdendritic areas, which results in dendritic areas rafting faster. Another aspect here may be the misfit δ, which differs in dendritic and interdendritic areas and is often more negative in the former than in the latter. At high temperatures, the misfit also becomes negative in the interdendritic areas, but the difference remains. Since areas with low misfit raft more slowly than areas with higher misfit, different characteristics of the raft structure can also occur here. Especially in the above-mentioned border areas at the fracture edge (see Figure 16a), isolated cube-like structures can still be observed, which indicate poor cross-linking. Considering the results of Yu et al. [27], it therefore seems possible that the retarded raft formation process on the one hand and the raft formation across a small-angle grain boundary hindered by the misorientation on the other hand lead to the fact that the connection to the neighbouring area is virtually non-existent. This consideration would also explain the results in Figure 15, as a combination of these two effects is conceivable on a larger length scale.

In summary, this means that the influence of interdendritic defects and areas with poor or no connection have an enormous impact on the strength of membranes when loaded parallel to the raft structure, i.e., perpendicular to the interdendritic regions, as these areas represent favoured fracture paths. However, γ′-bands with good connection on one side represent weak points in the microstructure, with the least influence according to the findings made here. Additionally, large-angle grain boundaries due to stray grains are preferred paths for crack propagation. However, their influence on the strength is comparatively small.

## 5. Conclusions

This research focussed on the relationship between the membrane microstructure and the mechanical characteristics. For this purpose, a raft structure was first obtained in a creep test at 950 °C and a stress of 250 MPa. A total of four creep states with creep strains between 1.3% and 5.7% and pore channel widths between 134 and 174 nm were produced. Selective phase extraction was used to dissolve the γ-matrix, creating a membrane structure from the remaining γ′-phase. In subsequent tensile tests, the mechanical properties were determined under perpendicular and parallel loading. A fracture surface analysis and additional EBSD measurements were then used to discuss the mechanical properties, which can be summarised as follows:

1. The mechanical strength of nanomembranes strongly depends on the cross-linking of the γ′-rafting structure. The creep strain plays an important role, generally leading to higher tensile strengths during perpendicular loading of the raft structure with increasing creep strain due to a continuous cross-linking process. The orientation of the fractured rafts itself indicates that the failure of the rafts is mainly caused by shear stresses.
2. The mechanical strength of nanomembranes during parallel loading of the raft structure strongly depends on the homogeneity of the microstructure. Orientation measurements using EBSD indicate that the formation of γ′-inhomogeneities originates from local orientation deviations between dendrites. The γ′-inhomogeneities can reduce the membrane strength due to a reduced cross-linking between areas with orientation deviations, leading to preferred crack progression during tensile testing.
3. In the context of the later premix membrane emulsification application, a creep strain of approximately 4% combines a good mechanical strength with a small pore size in both dendritic and interdendritic areas.

## Figures and Tables

**Table 1:** Summary of the single crystals used in this study and the corresponding experimental parameters in the creep test. * Note that the single crystal with the Specimen-ID CMSX-4/5.7/347 has a stray grain.

Specimen-ID	Cross Section/mm^2^	Stress *σ*/MPa	Creep Strain *ε_pl_*/%	Duration/h
CMSX-4/1.3/212	112.0	250	1.3	212
CMSX-4/2.7/282	112.0		2.7	282
CMSX-4/3.9/395	100.8		3.9	395
CMSX-4/5.7/347 *	112.0		5.7	347

**Table 2:** Results of the analysis of the pore structures from creep tests at 950 °C and 250 MPa using the line intersection method. Values for the tested membranes are given in nm for each dendritic (d) and interdendritic (id) areas (standard deviation in brackets). a refers to the vertical axis; c refers to the horizontal.

	CMSX-4/1.3/212	CMSX-4/2.7/282	CMSX-4/3.9/395	CMSX-4/5.7/347
c_d,γ-channel_	379 (310)	376 (273)	427 (351)	358 (270)
c_id,γ-channel_	341 (298)	361 (270)	411 (348)	423 (336)
a_d,γ-channel_	142 (44)	134 (42)	134 (39)	174 (55)
a_id,γ-channel_	141 (48)	156 (58)	140 (44)	204 (65)
c_d,γ′-ligament_	726 (596)	921 (725)	904 (755)	794 (588)
c_id,γ′-ligament_	733 (623)	805 (599)	1063 (857)	750 (596)
a_d,γ′-ligament_	280 (128)	350 (170)	300 (146)	400 (212)
a_id,γ‘-ligament_	318 (158)	360 (191)	383 (191)	383 (206)
(c/a)_d,γ-channel_	2.67	2.80	3.19	2.06
(c/a)_id,γ-channel_	2.42	2.31	2.94	2.07
(c/a)_d,γ′-ligament_	2.59	2.63	3.01	1.99
(c/a)_id,γ′-ligament_	2.31	2.24	2.78	1.96

**Table 3:** Results of the tensile tests for the load direction perpendicular to the raft structure (SD, standard deviation).

Specimen-ID	Creep Strain *ε_pl_*/%	Thickness *t*/mm	Width *w*/mm	Cross-Sectional Area/mm^2^	*R_m_*/ MPa	Mean *R_m_*/ MPa	SD *R_m_*/ MPa	E*/ GPa	Mean E*/ GPa	SD E*/ GPa	Mean *A*/%	Stdv. *A*/%
CMSX-4/1.3/212-1	1.3	0.30	10	3.0	52	72.75	15.75	8.3	8.9	0.9	0.63	0.01
CMSX-4/1.3/212-3		0.29	10	2.9	85			9.6			0.91	0.01
CMSX-4/1.3/212-5		0.29	10	2.9	85			9.8			0.88	0.01
CMSX-4/1.3/212-9		0.30	10	3.0	69			8.1			0.85	0.01
CMSX-4/2.7/282-3	2.7	0.30	10	3.0	82	93.00	9.55	13.6	13.9	0.7	0.60	0.00
CMSX-4/2.7/282-5		0.30	10	3.0	97			13.4			0.74	0.01
CMSX-4/2.7/282-7		0.30	10	3.0	104			14.9			0.71	0.00
CMSX-4/2.7/282-9		0.29	10	2.9	89			13.8			0.79	0.01
CMSX-4/3.9/395-1	3.9	0.30	10	3.0	104	106.25	5.12	11.5	11.6	0.3	0.93	0.01
CMSX-4/3.9/395-3		0.30	10	3.0	101			11.8			0.88	0.01
CMSX-4/3.9/395-5		0.30	10	3.0	107			11.2			0.99	0.01
CMSX-4/3.9/395-7		0.30	10	3.0	113			11.8			1.00	0.01
CMSX-4/5.7/347-1	5.7	0.30	10	3.0	137	118.75	17.34	16.7	15.9	0.8	0.84	0.00
CMSX-4/5.7/347-3		0.28	10	2.8	106			15.8			0.68	0.01
CMSX-4/5.7/347-5		0.29	10	2.9	102			16.1			0.64	0.00
CMSX-4/5.7/347-7		0.28	10	2.8	130			14.9			0.89	0.01

**Table 4:** Specifications of the round specimens used in the tensile test for the load direction parallel to the raft structure. The width and thickness at the fracture surface was measured using SEM.

Specimen-ID	Creep Strain *ε_pl_*/%	Thickness *t*/mm	Min. Width *w_min_*_/_mm	Max. Width *w_max_*/mm	*R_m_*_,*min*_/MPa	*R_m_*_,*max*_/MPa
CMSX-4/1.3/212-4-R	1.3	0.33	12.02	15.17	61	77
CMSX-4/1.3/212-6-LM	1.3	0.28	8.23	14.76	160	288
CMSX-4/2.7/282-4-RM	2.7	0.33	14.03	15.26	18	20
CMSX-4/3.9/395-4-RM	3.9	0.29	12.97	14.38	39	43
CMSX-4/5.7/347-8-LM	5.7	0.30	13.80	15.03	92	101
CMSX-4/5.7/347-8-L	5.7	0.30	14.18	14.58	62	63

## Data Availability

The original contributions presented in this study are included in the article. Further inquiries can be directed to the corresponding author.
